# Lexical and phraseological differences between second language written and spoken opinion responses

**DOI:** 10.3389/fpsyg.2023.1068685

**Published:** 2023-03-01

**Authors:** Minkyung Kim, Scott A. Crossley

**Affiliations:** ^1^Department of English Education, Korea National University of Education, Cheongju, Republic of Korea; ^2^Nagoya University of Commerce and Business, Nisshin, Japan; ^3^LEAR Laboratory, Department of Special Education, Vanderbilt University, Nashville, TN, United States

**Keywords:** modality, L2 written production, L2 spoken production, lexical complexity, phraseological complexity

## Abstract

This study examines differences in lexical and phraseological complexity features between second language (L2) written and spoken opinion responses *via* classification analysis. The study further examines the characteristics of L2 written and spoken responses that were misclassified in terms of lexical and phraseological differences, L2 learners’ vocabulary knowledge, and raters’ judgments of L2 use. The goal is to more thoroughly explore potential differences in lexical and phraseological production based on modality. The results indicated that L2 written responses tended to elicit greater lexical and phraseological complexity. The results also indicated that crossing the boundaries from L2 spoken to written (i.e., the use of less lexical and phraseological complexity) was related to lower levels of L2 vocabulary knowledge and tended to be penalized by raters in terms of L2 use. In contrast, crossing the boundaries from L2 written output to spoken (i.e., the use of greater lexical and phraseological complexity) was acceptable in terms of L2 use. Overall, this study highlights lexical and phraseological differences and the importance of the use of greater lexical and phraseological complexity in a modality-insensitive manner in L2 opinion-giving responses.

## 1. Introduction

One of the goals of learning a second language (L2) is successful engagement in L2 written and spoken communication. In this respect, a key agenda in L2 teaching and assessment is to develop and evaluate L2 learners’ ability to produce L2 written and spoken output for successful communication. Widely adopted communicative approaches are task-based language teaching and Common European Framework of Reference for Languages (CEFR)-based assessment, which both focus on meaning or communication, and L2 learners’ use of their own resources while carrying out production tasks (Council of Europe, 2001; Ellis, 2009; Skehan, 2018).^1^ Consequently, investigating L2 written and spoken production has been an important research topic in L2 research.

Over the past three decades, considerable attention has focused on how L2 learner output differs across modalities (i.e., writing and speaking modalities) based on task-based approaches using experimental approaches (e.g., Ellis and Yuan, 2005; Granfeldt, 2008; Kormos, 2014; Vasylets et al., 2017, 2019; Zalbidea, 2017) and from assessment perspectives using language corpora (e.g., Yu, 2010; Biber et al., 2016). These studies generally report that L2 writing tasks, as compared to speaking tasks, tend to elicit higher levels of linguistic complexity, defined as “the complexity directly arising from the number of linguistic elements and their interrelationships” (Pallotti, 2015, p. 117), particularly in terms of lexical and syntactic complexity. Phraseological complexity, an important element of L2 output (e.g., Paquot, 2019), however, has not yet been examined when comparing L2 writing and speaking tasks. Furthermore, there is a scarcity of research that has examined potential causes and consequences of producing L2 written responses that are characteristic of spoken responses and *vice-versa*.

To fill these gaps, this study examines differences in lexical and phraseological features between L2 written and spoken opinion responses *via* classification analysis. As well, the study investigates the characteristics of L2 written and spoken responses that are misclassified in terms of lexical and phraseological differences, L2 learners’ vocabulary knowledge, and raters’ evaluation of L2 use. The findings of this study provide comprehensive information concerning how modality influences L2 lexical and phraseological production in relation to learners’ L2 vocabulary knowledge and raters’ evaluation of L2 use.

## 2. Literature review

### 2.1. Psycholinguistic processes in writing and speaking

To examine similarities and differences in lexical and phraseological features across modalities, it is important to discuss the psycholinguistic mechanisms involved in speaking and writing. The current influential theoretical models of both writing (e.g., Hayes and Berninger, 2014) and speaking (e.g., Levelt, 1992) in the L1 literature postulate four similar stages: (1) proposal and conceptualization, (2) translation and formulation, (3) transcription and articulation, and (4) evaluation and self-monitoring in writing production (Hayes and Berninger, 2014) and speech production (Levelt, 1992), respectively. The proposal/conceptualization stage involves generating the message (i.e., what writers and speakers want to convey). The translation/formulation stage involves transforming the conceptual message into language strings of verbal forms *via* lexical selection and grammatical encoding. The transcription/articulation stage involves producing written symbols in writing and speech sounds in speaking. Finally, the evaluation/self-monitoring stage concerns checking the appropriateness of the output. These stages posited for L1 written and oral production are considered applicable to L2 production (e.g., Kormos, 2006; Kim et al., 2021).

When individuals carry out similar tasks in writing and speaking, their ability to select lexical information and retrieve a sequence of words likely impacts the translation and formulation processes in writing and speaking, respectively, in a way that higher levels of such ability likely lead to more efficient and automatic lexical processing (Levelt, 1992; Kormos, 2006; Hayes and Berninger, 2014; Tavakoli, 2014; Skehan, 2018). Thus, greater levels of L2 lexical and phraseological resources likely enhance lexical and phraseological encodings of the message for both writing and speaking, resulting in the better use of lexical and phraseological features found in the written and spoken output (Kormos, 2014).

While the global processes of written and oral production are similar to some degree, there are essential differences concerning L2 lexical and phraseological production, particularly in terms of the degree of time pressure and the nature of the output (Kormos, 2006; Williams, 2012; Tavakoli, 2014). Speaking imposes much greater online pressure due to time constraints than writing. The time pressure in speaking (as compared to writing) limits the processes of conceptualizing and formulating the message, constraining the access to existing language knowledge and the implementation of language production processes. This time pressure in speaking might be particularly greater for L2 learners whose language knowledge is limited and whose production processes are less automatic (Kormos, 2014; Skehan, 2018). On the other hand, writing offers more time for generating the message, translating the message into verbal forms, and revising and editing the output in a self-paced and recursive manner (Hayes and Berninger, 2014). Furthermore, the output of speaking is evanescent, while the output of writing is visible. The visibility of the written output potentially reduces the cognitive load, facilitates knowledge recall, and enhances the monitoring (including revising and editing) processes in writing (Grabowski, 2007; Williams, 2012). Taken together, the differences between oral and written production can lead to lexical and phraseological consequences, such that writing (which offers more time and access to visible text) potentially allows L2 leaners to produce more lexically and phraseologically complex features as compared to speaking. This assumption in relation to lexical complexity (not phraseological complexity) is supported by empirical studies.

### 2.2. Lexical complexity in writing and speaking

Many L2 studies have compared written and oral production in terms of lexical complexity, reporting consistent findings that writing tends to elicit higher lexical complexity than speaking (e.g., Ellis and Yuan, 2005; Granfeldt, 2008; Kormos, 2014; Biber et al., 2016;Vasylets et al., 2017, 2019; Zalbidea, 2017). Some of these studies have examined written and oral discourse produced by the same learners (Ellis and Yuan, 2005; Granfeldt, 2008; Kormos, 2014). For example, Ellis and Yuan (2005) sampled written and spoken narratives produced by the same 42 Chinese (L1) intermediate-level learners of English (L2) and reported greater lexical diversity (as measured by mean segmental type-token ratio) in writing than in speaking. Kormos (2014) examined written and spoken narratives produced by the same 44 Hungarian (L1) intermediate-level learners of English (L2) and found higher greater diversity (as measured by vocd-D) and lexical sophistication (as measured by lexical profile measures, i.e., greater use of low-frequency and academic words) in writing.

Other studies have compared writing and speaking using the same task but produced by different learners (Vasylets et al., 2017, 2019; Zalbidea, 2017). Zalbidea (2017) examined written emails and oral explanations (argumentative) produced by 16 English-speaking (L1) intermediate-level learners of Spanish (L2), respectively, and reported higher lexical diversity (measured by the Guiraud’s index) in writing than in speaking. Vasylets et al. (2019) focused on written and spoken narratives produced by 145 Spanish-speaking learners of English (L2) with mixed proficiency levels (from intermediate to advanced), respectively, and reported higher lexical density, lexical diversity (measured by vocd-D and Guiraud’s index), and lexical sophistication (measured by the Advanced Guiraud index; i.e., greater use of less frequent words) in writing than in speaking.

### 2.3. Phraseological complexity in writing and speaking

Comparing lexical complexity between L2 writing and speaking can provide important information concerning how single words are differently used across modalities. However, L2 production is not only lexically driven, but also phraseologically driven. That is, L2 production depends on L2 learners’ knowledge of not only individual vocabulary items but also “the sequential probabilities of linguistic elements” (Ellis et al., 2015, p. 358). Accordingly, phraseological features used by L2 learners likely differ across modality as lexical features do. However, to our knowledge, such assumption has not been tested yet.

Phraseological units (i.e., word combinations) have been widely researched using various terms including formulaic sequences, *n*-grams, lexical bundles, and collocations and employing various criteria in determining phraseological units including semantic transparency, frequency, and associative strength of multi-word units (Paquot and Granger, 2012; for a recent review, see Ebeling and Hasselgård, 2021). Paquot (2019) defines phraseological complexity as “the range of phraseological units that surface in language production and the degree of sophistication of such phraseological units” (p. 124). Regardless of the terms or criteria used, much research has provided evidence for the psycholinguistic reality of phraseological units, such that the more frequent and the more strongly associated a phraseological unit, the faster and the more accurately it is processed (e.g., Ellis et al., 2008; Ellis, 2009; for a review, see Siyanova-Chanturia and Van Lancker Sidtis, 2018).

One of the important dimensions in examining phraseological units is co-occurrence defined as “the preference that words have for certain co-occurring words” (Paquot and Granger, 2012, p. 136). In examining co-occurrence, the corpus-based approach to phraseology (Sinclair, 1991) has been widely adopted, which focuses on the strength of meaningful associations of phraseological units (i.e., above-chance co-occurrence) from a statistical perspective (rather than linguistic criteria; Boers and Webb, 2018). In this line of research, two well-established association strength measures include t-scores and mutual information (MI; Evert, 2009; Granger and Bestgen, 2014; Gablasova et al., 2017). These measures represent the probability that two lexical items co-occur and can be calculated using observed co-occurrence frequency and expected frequency of word pairs (see Evert, 2009 for calculation of t-scores and MI). T-scores tend to inflate high-frequency words that co-occur with various potential partner words (e.g., *he was, of the;* Evert, 2009). On the other hand, MI is known to inflate with low-frequency words that co-occur with the small number of potential partner words (i.e., rare combinations; e.g., *circumstances in which, of the court of appeal;* Ellis et al., 2008). Using association strength measures for phraseological units, previous L2 studies have found that L2 learners (as compared to L1 speakers) tend to overuse phraseological units with high t-scores and underuse phraseological units with high MI scores (e.g., Durrant and Schmitt, 2009). However, what is less known are differences in phraseological features between L2 written and spoken performances. Potentially, given that L1 speech is different from L1 writing in terms of the use of formulaic expressions (Biber et al., 1999), L2 written and spoken performances may also show differences in the use of phraseological units.

### 2.4. Lexical and phraseological complexity, L2 vocabulary knowledge, and rater evaluation

Many previous studies have investigated differences in lexical complexity between L2 writing and speaking leading to the prediction that there might also be differences in phraseological complexity between L2 writing and speaking. These differences may allow L2 writing and speaking responses to be successfully classified based on lexical and phraseological features. Additionally, the production of lexical and phraseological features may relate to learner variables, such as vocabulary knowledge, and may also cause misclassifications.

Previous research has explored potential influences in the production of lexical and phraseological features in L2 written and spoken responses, including L2 vocabulary knowledge (e.g., Laufer and Nation, 1995; Milton et al., 2010; Henriksen and Danelund, 2015; Uchihara and Clenton, 2020). With respect to the relationships between L2 vocabulary knowledge (which is commonly measured using a written test) and lexical features found in L2 learner production, different patterns have been reported depending on modality. For instance, research has indicated that the higher a learner’s L2 vocabulary knowledge, the greater lexical complexity features are produced in L2 writing (e.g., Laufer and Nation, 1995; Henriksen and Danelund, 2015). This is likely because larger L2 vocabulary may facilitate the retrieval and use of more various and sophisticated lexical items during L2 writing. In contrast, in L2 speaking research, weak associations between L2 vocabulary knowledge (when measured using a written form) and L2 lexical production have been reported (e.g., Milton et al., 2010; Uchihara and Clenton, 2020). This may be because the use of more complex lexical features may not be required for spoken discourse, particularly in casual conversation. To our knowledge, though, the relationship between L2 vocabulary knowledge and phraseological complexity has not been explored. However, we speculate that greater L2 vocabulary knowledge may help retrieve and select contextually appropriate phraseological units.

Previous studies have also examined the effects of lexical and phraseological features in L2 written and spoken responses on human ratings of L2 performances. Studies have reported that lexical complexity is predictive of L2 writing (e.g., Biber et al., 2016; Kyle and Crossley, 2016; Kim et al., 2018) and L2 speaking (e.g., Crossley et al., 2011; Eguchi and Kyle, 2020), and that phraseological complexity (when measured by MI and not t-scores) is predictive of L2 writing (e.g., Bestgen and Granger, 2018; Kim et al., 2018; Garner et al., 2019; Paquot, 2019) and L2 speaking (e.g., Eguchi and Kyle, 2020; Saito, 2020; Uchihara et al., 2021). Findings from these studies suggest that producing more complex lexical units and more strongly associated phraseological units is associated with better L2 written and spoken performances.

Building on these previous studies, we investigate the characteristics of L2 written and spoken responses that are both correctly classified and those that are misclassified based on lexical and phraseological differences, L2 learners’ vocabulary knowledge, and raters’ evaluation of L2 use. Note that we examine L2 vocabulary knowledge and raters’ evaluations of L2 use in terms of the view that misclassified L2 written and spoken responses may be related to L2 learners’ vocabulary knowledge and raters’ evaluation of L2 use.

### 2.5. Current study

Previous research has examined differences in lexical complexity features between L2 written and spoken performance. In addition to lexical features, phraseological features have also been an important component in L2 research. To our knowledge, however, few if any studies have examined potential differences in phraseological features between L2 written and spoken performances. In addition, little is known about the characteristics of L2 written responses that have more oral characteristics, and those of L2 spoken responses that have more written characteristics. Biber et al. (2016) hinted at advantages of L2 spoken responses having written characteristics in terms of rater evaluation. Furthermore, for L2 written and spoken response that have stronger oral and written characteristics, respectively, little is known about how those responses’ features may relate to L2 learners’ vocabulary knowledge and/or raters’ evaluation of L2 use. For example, L2 written responses that have more oral characteristics may be related to limited vocabulary knowledge on the part of the writer and may lead to lower human rating of L2 use. With this in mind, the current study was guided by the following two research questions:

1. To what extent can lexical and phraseological features classify L2 written and spoken opinion responses?
2. For responses that are misclassified, do their characteristics in terms of lexical and phraseological features, L2 learners’ vocabulary knowledge, and raters’ evaluation of L2 use help define the misclassifications?

## 3. Materials and methods

### 3.1. Corpus

We used L2 (English) writing and speaking performance data from test-takers on the Examination for the Certificate of Competency in English (ECCE), which is based on the CEFR. The ECCE, developed by Michigan Language Assessment, aims at assessing high-intermediate level English proficiency (i.e., the B2 level of the CEFR). We used a total of 238 test-takers who produced both writing and speaking samples (i.e., paired data) in response to the ECCE speaking and writing sections. For the 238 test-takers, 141 (59.243%) were female. The test-takers ranged in age from 13 to 47 with a mean of 19.224 (SD = 5.799). The test population consisted of test-takers whose L1s were Spanish (*n* = 166; 69.748%) and Portuguese (*n* = 72; 30.252%).

The writing section of the ECCE measures the ability to produce clear text. It asked test-takers to read a short excerpt from a newspaper article about a situation or issue (e.g., increasing the cost of tickets for the city’s professional soccer team) and then write a letter or essay giving an opinion about the situation or issue. Thus, there were two different genres in the written dataset: letters (e.g., “The City Times is interested in citizens’ opinions about the new supermarket. Do you think it should be built in your city or not? Write a letter to the editor, giving specific reasons to explain your view.”) and essays (e.g., “What are the advantages and disadvantages of shopping at very large supermarkets? How does it compare to shopping in small, local stores? Give specific examples to support your answer”). The current data set included four different writing prompts.^2^ Test-takers were provided 30 min to complete the writing section. Hand-written writing samples were scanned, and transcribed into electronic format. The writing dataset was balanced in terms of the two writing genres, such that it included 119 letter samples and 119 essay samples. Although the writing section had two different genres (i.e., letters and essays), we analyzed them together for three reasons. First, the same scoring rubric was used to rate letters and essays. Second, both letter prompts and essay prompts asked test-takers to provide an opinion on a controversial topic. Lastly, no significant differences between letters and essays were found in word counts [*t*(236) = 1.032, p = 0.303].

The speaking section of the ECCE measures the ability to speak in an interactive and fluent manner. In the speaking section, a structured interview between a test-taker and an examiner was conducted, which lasted about 15 min. The current data set included 20 different speaking prompts. The interview consisted of four sequential tasks, but we included test-takers’ responses to the third question of Task 4 only, which lasted around one or 2 min. The third question asked test-takers to provide an opinion on an issue (e.g., “Some people believe that public money should not be used for occasions like town anniversaries because such events do not directly benefit anyone. To what extent do you think this is true?”). We chose to analyze the third question of Task 4 because it involved a speaking component only (i.e., responding to the given question) and its task was similar to the writing section (i.e., giving an opinion). Each test-taker’s response was transcribed and each transcript was then cleaned to eliminate fillers (e.g., *um* and *er*) and interjections (e.g., *oh* and *ah*). We also deleted repetitions and false starts (e.g., *the, the, the, teacher* was modified to *the teacher*) so as not to include these repeated items in word counts.

### 3.2. Linguistic measures

We measured various lexical and phraseological complexity features using available NLP tools. The lexical and phraseological complexity features are discussed below.

#### 3.2.1. Lexical measures

Lexical complexity was measured in terms of lexical density, diversity, and sophistication. Lexical density was measured as the ratio of the number of content word tokens to the total number of tokens in a text (O'Dell et al., 2000). Lexical diversity was measured using the hypergeometric distribution diversity index (HD-D; McCarthy and Jarvis, 2007), which computes, for each word type in a text, the probability of finding one of its tokens in a random sample of 42 tokens. The sum of the probabilities for all lexical types in the text is used as the HD-D value for the text. Lexical density and HD-D were computed using the tool for the automatic analysis of lexical diversity (TAALED; version 1.4.1; see Kyle et al., 2021 for more information on TAALED). The TAALED converts HD-D to the same scale as a type-token ratio for ease of interpretation.

Lexical sophistication was measured using word frequency and age of acquisition, which are often employed as proxies for word difficulty (e.g., Brysbaert et al., 2019). Generally, low frequency words and words acquired at a later age are considered more difficult, sophisticated words. Lexical sophistication for functions words was not considered because function words tend to have a nonconceptual meaning, fulfilling a grammatical-syntactic function, rather than carrying specific semantic content (O'Dell et al., 2000). In addition, to measure lexical sophistication, lemmas (i.e., base forms of words, such as *look* for *looked* and *looks*) were used because we presumed that adding inflections to base forms would not contribute to lexical sophistication. Thus, through lemmatization, the same lexical sophistication values were assigned to base forms and their inflected forms. To control for the potential effects of prompt words on lexical use during speaking and writing performance, content words (CW; lemmas) that appeared in a prompt were removed from each sample produced for that prompt.

Word frequency was measured based on the spoken and academic subsections of the Corpus of Contemporary American English (COCA; Davies, 2009). Being aware of differences in lexical features between spoken and written discourses (Dang et al., 2017), we chose both of the spoken and academic subsections. We further assumed that frequency indices based on the spoken and academic sections might be more closely related to L2 learners’ speaking and writing production (i.e., opinion responses), respectively. Frequency scores were expressed as log-transformed scores.

Age-of-acquisition scores were calculated based on Kuperman et al. (2012), which are expressed as the mean ages in years at which native speakers of English thought they had acquired the word. A majority of the age-of-acquisition ratings ranges from ages five to 14. Considering that the age-of-acquisition effects (i.e., earlier acquired, more quickly processed) in an L1 tend to be applied to L2 speakers to some degree (e.g., Izura et al., 2011), words learned at a later age by L1 speakers may also be considered more difficult by L2 learners.

The frequency/age-of-acquisition scores of each sample were calculated using the tool for the automatic analysis of lexical sophistication (TAALES; version 2.2; see Kyle et al. (2018) for more information on TAALES) as the mean frequency/age-of-acquisition score by dividing the sum of the frequency/age-of-acquisition scores for the lemmas in a sample by the number of lemmas in that sample that received scores. Lemmas not found in the frequency/age-of-acquisition word list were not counted toward the mean scores.

#### 3.2.2. Phraseological measures

Phraseological complexity was measured using two bigram association strength measures: t-and MI scores. When association strength of bigrams was calculated, we included all of bigrams regardless of parts of speech of the words that composed bigrams, following previous studies (Granger and Bestgen, 2014; Bestgen and Granger, 2018; Saito and Liu, 2022). We chose t-and MI scores because they have been widely examined in L2 studies (e.g., Granger and Bestgen, 2014; Kim et al., 2018; Garner et al., 2019; Paquot, 2019; Saito and Liu, 2022), but differences in the use of *n*-grams between L2 writing and speaking performance are less known.

T-scores were calculated as “the observed frequency minus the expected frequency divided by the square root of the observed frequency,” and MI scores were calculated as “the logarithm of the observed co-occurrence of two items divided by the expected co-occurrence of two items” (Kyle et al., 2018, p. 1036; also see Kyle et al., 2018 for more information on MI and t-scores). T-scores tend to highlight frequent items with *n*-grams that consist of higher-frequency words receiving higher t-scores, whereas MI scores tend to highlight the importance of low-frequency items with *n*-grams that consist of lower-frequency words receiving higher MI scores (Evert, 2009). All of the bigram indices were based on both of the spoken and academic sections of COCA and calculated using the TAALES (version 2.2; Kyle et al., 2018). Bigrams not found in the TAALES *n*-gram lists were not counted toward the mean scores.

### 3.3. L2 vocabulary knowledge

L2 vocabulary knowledge was measured by the vocabulary subsection of the Grammar/Vocabulary/Reading section of the ECCE. Given that the ECCE was developed based on the CEFR, the vocabulary subsection related to measuring linguistic competence (including lexical, phonological, syntactical knowledge and skills) which is part of the communicative language competence outlined in the CEFR (Council of Europe, 2001, p. 13). L2 vocabulary knowledge was operationalized as the ability to identify appropriate vocabulary units at the sentential level. The vocabulary items consisting of multiple-choice items asked test-takers to complete a sentence (e.g., “Everyone thought that the new student was a welcome _to the class.”) by selecting one of the four options that best completes the sentence (e.g., “increase, growth, development, and addition”). The four options shared the same part of speech. To answer each question, test-takers were expected to understand the context (i.e., the sentence) in which the target word occurred. Test-takers were given 90 min to complete the entire GVR section, and, thus, no specific time limit was set for the vocabulary subsection. The maximum possible score for the vocabulary test used for this study was 26. The Cronbach’s alpha for the 26 items was 0.74.

### 3.4. L2 use scores

#### 3.4.1. Language use during L2 writing

Each writing sample was rated using an analytic five-point rating scale with four criteria (i.e., *content and development*, *organization and connection of ideas*, *linguistic range and control*, and *communicative effect*). The writing samples were independently scored by two expert raters. If two raters had nonadjacent scores for a writing sample, a third rater evaluated it (Michigan Language Assessment, 2017). Average scores were calculated, and used in this study. While inter-rater reliability scores were unavailable for the data used in this study, the overall within tolerance agreement (±2 score points) percentage was 84.24%, which was considered reasonably high (Michigan Language Assessment, 2017). For this study, we used scores for a criterion for an L2 writing opinion task: *linguistic range and control*, which concerned variety and precision of grammar and vocabulary during L2 writing. Scores of *linguistic range and control* for the writing section were used as L2 use scores for the L2 writing opinion task.

#### 3.4.2. Language use during L2 speaking

The language use during L2 speaking performance was quantified based on scores in the speaking section of the ECCE. The speaking scores were rated by the interviewer (examiner), using an analytic five-point rating scale. The scoring rubric had three criteria for each speaking task: *overall communicative effectiveness, language control and resources, and delivery and intelligibility.* In this study, we used a criterion of *language control and resources*, which concerned the use of linguistic resources including grammar and vocabulary during L2 speaking. These scores were based on test-takers’ performances on all of the three questions of Task 4. Thus, one caveat is that scores of *language control and resources*, which were used as L2 use scores for the L2 speaking opinion task in this study, reflected language use for not only the third question (i.e., the opinion question) but also the other two questions of Task 4.

### 3.5. Analysis

To answer research question 1 (i.e., to what extent lexical and phraseological features found in L2 written and spoken opinion responses can classify writing and speaking samples), we first conducted paired t-tests and then constructed a generalized linear mixed model (GLMM). Using a 67/33 split, the data were divided into a training set and a test set. Average L2 use scores for both writing and speaking tasks in the training sets were similar to those in the test sets. To select lexical and phraseological features that showed significant differences between written and spoken responses, using the training set, we conducted paired *t*-tests, and, if required because of violations of normal distribution, non-parametric Wilcoxon signed-rank tests (Larson-Hall, 2015). The distributions of the differences were checked through visual inspection and levels of skewness and kurtosis. The values for skewness and kurtosis between −2 and + 2 were considered acceptable to indicate a shape close to normal distribution (George and Mallery, 2010). To mitigate the possibility of increases in the type I error (the false positive) due to multiple comparisons, Bonferroni corrections were used. An alpha was set at 0.017 (0.05/3) to compare the three measures of CW lexical sophistication, and 0.013 (0.05/4) to compare the four measures of bigram t-and MI scores.

A GLMM then was constructed using variables selected from the t-test analysis as predictors. The GLMM was created using the training set (*n* = 318), and then applied into the test set (*n* = 158) to evaluate how well the model classified writing and speaking samples in a new dataset. GLMMs address linear mixed models (that include both fixed and random effects) and generalized linear models (that handle non-normal data, such as binomial distributions) to develop a classification model (Faraway, 2016). In our GLMM model, the response variable was a binomial response defined as either spoken (coded as 0) or written responses (coded as 1). The fixed effects were the lexical and phraseological features that showed significant differences between written and spoken responses with at least small effect sizes (based on the results of *t*-tests or Wilcoxon signed-rank tests). Among these features, when multicollinearity (defined as *r* > 0.699) was detected, the feature that showed the largest effect size was retained while the other feature was removed. Random effects in GLMM quantify variation across participants. The GLMM model created for this study used backward selection of the fixed effects, such that only significant fixed effects (*t* > 1.96 at a 0.05 significance level) were retained. We did not consider interaction effects or random slopes because certain levels (however small) of interactions between the lexical and phraseological feature were expected given that the features were measured on the same text. In addition, a random slope model could not be estimated because the amount of repeated-measurements structure was minimal (the participants provided two data points only).

Building on the GLMM results, we investigated L2 written responses classified as spoken responses and spoken responses classified as written responses (research question 2) in terms of lexical and phraseological features (which were included as significant predictors in the GLMM), L2 vocabulary test scores, and L2 use scores. To do so, we conducted Welch’s independent t-tests, which are considered more reliable when the two samples have unequal variances or unequal sample sizes (Levshina, 2015). If required because of violations of normal distribution, we also conducted non-parametric Wilcoxon rank sum tests.

For the analyzes related to comparing paired or unpaired two groups, effect sizes were reported. Cohen’s *d* was used as an effect size statistic for *t*-tests (Cohen, 1988): small (0.2 ≤ *d* < 0.5), medium (0.5 ≤ *d* < 0.8), and large (*d* ≥ 0.8). The correlation coefficient *r* was used as an effect size statistic for Wilcoxon signed-rank tests and Wilcoxon rank sum tests (*r* was calculated as the absolute standardized statistic *z* divided by square root of the total number of pairs, which interpretation coincides with that for Pearson’s correlation coefficient; Tomczak and Tomczak, 2014): small (0.1 ≤ *r* or *rho* < 0.3), medium (0.3 ≤ *r* or *rho* < 0.5), and large (*r* or *rho* ≥ 0.5). When results did not reach statistical significance, we did not calculate effect sizes. When the relationships did not reach small effects, even though they showed statistical significance, they were not further considered because negligible effect sizes are considered less meaningful (Sullivan and Feinn, 2012). All statistical analyzes were performed with *R* (R Core Team, 2020).

## 4. Results

### 4.1. Descriptive statistics

Descriptive statistics of lexical and phraseological features are provided in Table 1. While the average number of word counts in L2 written responses [*M* (SD) = 212.803 (46.917)] was greater than that of L2 spoken responses [*M* (SD)= 103.584 (43.898); *t*(237) = −28.400, *p* < 0.001, *d* = 1.840], it should be noted that all word and phraseological count features are normed for text length.

**Table 1:** Descriptive statistics for lexical and phraseological features found in L2 written and spoken responses.

Variable	Writing mean (SD)	Speaking mean (SD)
Lexical density	0.441 (0.038)	0.386 (0.045)
HD-D	0.787 (0.033)	0.710 (0.048)
COCA academic frequency CW (log, no prompt words)	2.472 (0.144)	2.507 (0.150)
COCA spoken frequency CW (log, no prompt words)	2.538 (0.169)	2.767 (0.195)
Age of acquisition CW (no prompt words)	5.637 (0.367)	5.195 (0.430)
COCA academic bigram T	38.598 (9.904)	27.930 (29.170)
COCA spoken bigram T	58.955 (10.726)	68.226 (20.576)
COCA academic bigram MI	1.563 (0.171)	1.477 (0.268)
COCA spoken bigram MI	1.600 (0.161)	1.427 (0.214)

### 4.2. Research question 1

To investigate whether lexical and phraseological features found in L2 written and spoken opinion responses produced by the same L2 learners could distinguish writing and speaking samples (research question 1), we first conducted *t*-tests, and Wilcoxon signed-rank tests (if required due to violations of normality) using the training set (*n* = 318). The results are provided in Table 2. Significant differences with at least small effects between L2 written and spoken opinion responses were found in all of the 9 lexical and phraseological features.

**Table 2:** *T* statistics for *t*-test results (or *z* statistics for Wilcoxon signed-rank test results) for lexical and phraseological features between L2 written and spoken responses in the training set (*n* = 318).

Variable	*t* or *z* statistic	*p*	Effect size (*d* or *r*)
Lexical density	*t* = −11.920	<0.001	*d* = 0.945
HD-D	*t* = −16.090	<0.001	*d* = 1.28
COCA academic frequency CW (log, no prompt words)	*t* = 2.899	0.004	*d* = 0.230
COCA spoken frequency CW (log, no prompt words)	*t* = 12.015	<0.001	*d* = 0.953
Age of acquisition CW (no prompt words)	*t* = −10.91	<0.001	*d* = 0.865
COCA academic bigram T	*z* = −4.448	<0.001	*r* = 0.353
COCA spoken bigram T	*t* = 6.008	<0.001	*d* = 0.476
COCA academic bigram MI	*t* = −3.031	0.003	*d* = 0.240
COCA spoken bigram MI	*t* = −8.351	<0.001	*d* = 0.662

Based on the t-test results, we constructed a GLMM using the training set. Among the nine lexical and phraseological features that showed significant differences, one feature (i.e., COCA academic bigram MI) was strongly correlated with another (i.e., COCA spoken bigram MI; *r* = 0.775), and was removed to control for multicollinearity. Next, to construct a baseline GLMM, using the training set, a random intercept model was created including the participants as random intercepts. However, this model did not explain any variance in classifying written and spoken responses. Performing backward selection of fixed effects, the GLMM included six significant variables (see Table 3). The results indicate that L2 written opinion responses (as compared to L2 spoken opinion responses) were characterized by greater lexical diversity (as measured by HD-D; *z* = 4.650, *p* < 0.001), greater association strength of bigrams consisting of high-frequency words (as indicated by higher bigram t-scores) based on the academic corpus (*z* = 4.442, *p* < 0.001), greater CW sophistication (as indicated by CW age of acquisition; *z* = 3.646, *p* < 0.001), greater lexical density (*z* = 3.615, *p* < 0.001), and greater association strength of bigrams consisting of low-frequency words (as indicated by bigram MI) based on the spoken corpus (*z* = 1.363, *p* < 0.001). On the other hand, L2 spoken opinion responses (as composed to L2 written opinion responses) featured greater association strength of bigrams consisting of high-frequency words (as indicated by higher bigram t-scores) based on the spoken corpus (*z =* −4.463, *p* < 0.001). The GLMM model explained 86.287% of the variance using the fixed factors, while no variance was explained by random effects.

**Table 3:** Results of the generalized linear mixed model (GLMM) to classify L2 written and spoken responses.

Fixed effect	Estimate	Standard error	*z*	*p*
(Intercept)	−42.039	6.242	−6.734	<0.001
HD-D	29.631	6.373	4.650	<0.001
COCA spoken bigram T	−0.108	0.024	−4.463	<0.001
COCA academic bigram T	0.124	0.028	4.442	<0.001
Age of acquisition CW (no prompt words)	1.870	0.513	3.646	<0.001
Lexical density	17.752	4.910	3.615	<0.001
COCA spoken bigram MI	3.127	1.323	2.363	<0.05

The GLMM was then used to investigate how accurately it classified L2 written and spoken responses in the training set (see Table 4). The GLMM correctly allocated 289 of the 318 samples in the training set for an accuracy of 90.881%. The GLMM classification model was extended to the test set to assess classification accuracy in a new dataset (see Table 5). The GLMM correctly allocated 145 of the 158 samples in the test set for an accuracy of 91.772%. These results provide strong evidence that lexical and phraseological features as found in L2 written and spoken responses can successfully classify L2 written and spoken responses, highlighting lexical and phraseological differences specific to written and spoken opinion responses produced by the same L2 learners.

**Table 4:** Confusion matrix for classifying writing and speaking in the training set.

Actual group	Predicted group	
	Spoken response	Written response
Spoken response	144	15
Written response	14	145

Accuracy = 0.909; Precision = 0.906; recall = 0.912; F1 = 0.909.

**Table 5:** Confusion matrix for classifying writing and speaking in the test set.

Actual group	Predicted group	
	Spoken response	Written response
Spoken response	71	8
Written response	5	74

Accuracy = 0.918; Precision = 0.902; recall = 0.937; *F*1 = 0.919.

### 4.3. Research question 2

Building on the GLMM results, we explored the characteristics of L2 written responses misclassified as L2 spoken responses. Specifically, we examined responses misclassified in both the training (*n* = 14) and test (*n* = 5) sets in terms of lexical and phraseological features (which were included as significant predictors in the GLMM), L2 vocabulary test scores, and L2 use scores. We compared them against L2 written responses correctly classified in the training (*n* = 145) and test (*n* = 74) sets. The results of the t-tests are provided in Table 6. The t-test results indicated that L2 written responses classified as spoken showed lower lexical diversity (as measured by HD-D; *t* = 7.700, *p* < 0.001), lower CW sophistication (as indicated by CW age of acquisition; *t* = 5.666, *p* < 0.001), lower lexical density (*t* = 3.750, *p* < 0.01), and lower association strength of bigrams consisting of low-frequency words (as indicated by bigram MI based on the spoken corpus; *t* = 5.646, *p* < 0.001). In addition, L2 learners whose written responses were classified as spoken tended to receive lower L2 vocabulary test scores (*t* = 2.415, *p* < 0.05) and lower L2 use scores in their written responses (*t* = 2.117, *p* < 0.05) than those whose written responses were classified as written ones.

**Table 6:** *t* statistics for *t*-test results between L2 written responses classified as spoken ones and those classified as written ones.

Variable	Mean (*SD*) of L2 written responses classified as spoken (*n* = 19)	Mean (*SD*) of L2 written responses classified as written (*n* = 219)	*t*	*p*	Effect size (*d*)
HD-D	0.740 (0.028)	**0.791** (0.031)	7.700	<0.001	1.760
COCA spoken bigram T	63.585 (11.389)	58.553 (10.599)	−1.858	0.077	Not applicable
COCA academic bigram T	34.530 (10.745)	38.951 (9.774)	1.733	0.098	Not applicable
Age of acquisition CW (no prompt words)	5.305 (0.220)	**5.666** (0.363)	6.449	<0.001	1.200
Lexical density	0.410 (0.038)	**0.444** (0.037)	3.750	<0.01	0.910
COCA spoken bigram MI	1.417 (0.146)	**1.615** (0.153)	5.646	<0.001	1.230
L2 vocabulary test scores	19.526 (3.116)	**21.342** (3.386)	2.415	<0.05	0.558
L2 use score	3.184 (0.533)	**3.461** (0.694)	2.117	<0.05	0.448

Significant higher scores between the two groups were in bold.

We also explored the characteristics of L2 spoken responses classified as L2 written responses. Specifically, we investigated responses misclassified both in the training (*n* = 15) and test (*n* = 8) sets in terms of lexical and phraseological features (which were included as significant predictors in the GLMM), L2 vocabulary test scores, and L2 use scores. We compared them against L2 spoken responses correctly classified as spoken in both the training (*n* = 144) and test (*n* = 71) sets. The results of the t-tests (and Wilcoxon rank sum test results) are provided in Table 7. The results indicated that L2 spoken responses classified as written showed greater lexical diversity (as measured by HD-D; *t* = −6.988, *p* < 0.001), lower association strength of bigrams consisting of high-frequency words (as indicated by bigram t-scores) based on the spoken corpus (*t* = 3.237, *p* < 0.01), greater CW sophistication (as indicated by CW age of acquisition; *t* = −5.230, *p* < 0.001), and greater lexical density (*t* = −4.415, *p* < 0.01). No differences were reported in L2 vocabulary test scores or L2 use scores.

**Table 7:** *t* statistics for *t*-test results (or *z* statistics for Wilcoxon rank sum test results) between L2 spoken responses classified as spoken ones and those classified as spoken ones.

Variable	Mean (SD) of L2 spoken responses classified as written ones (*n* = 23)	Mean (SD) of L2 spoken responses classified as spoken ones (*n* = 215)	*t* or *z*	*p*	Effect size (*d* or *z*)
HD-D	**0.763** (0.038)	0.704 (0.046)	*t* = −6.988	<0.001	*d* = 1.410
COCA spoken bigram T	56.625 (17.807)	**69.467** (20.501)	*t* = 3.237	<0.01	*d* = 0.669
COCA academic bigram T	36.847 (12.478)	26.976 (30.280)	*z* = −1.956	0.050	Not applicable
Age of acquisition CW (no prompt words)	**5.570** (0.355)	5.155 (0.419)	*t* = −5.230	<0.001	*d* = 1.070
Lexical density	**0.423** (0.043)	0.382 (0.044)	*t* = −4.314	<0.001	*d* = 0.938
COCA spoken bigram MI	1.458 (0.218)	1.423 (0.213)	*t* = −0.718	0.479	Not applicable
L2 vocabulary test scores	22.391 (3.539)	21.070 (3.363)	*t* = −1.710	0.099	Not applicable
L2 use score	3.739 (0.915)	3.670 (0.847)	*t* = −0.348	0.731	Not applicable

Significant higher scores between the two groups were in bold.

## 5. Discussion

### 5.1. Research question 1: Classification of L2 written and spoken opinion responses using lexical and phraseological complexity features

Research Question 1 examined whether lexical and phraseological complexity features found in L2 written and spoken opinion responses produced by the same L2 learners could distinguish L2 written and spoken opinion responses. We first conducted *t*-tests to compare lexical and phraseological features between L2 written and spoken output. In terms of lexical complexity features, consistent patterns were observed, such that L2 written responses featured greater lexical complexity as indicated by greater levels in lexical density, lexical diversity, and lexical sophistication measures than L2 spoken responses.

With respect to phraseological complexity features, different patterns were found depending on phraseological measures used. For bigram MI scores, L2 written responses consistently showed higher MI scores (which tend to inflate with low-frequency word combinations) than L2 spoken responses when measured based on both academic and spoken corpora. This was partly because bigrams that received higher MI scores based on the academic corpus (e.g., *global warming, vast majority*, and *human beings*) also tended to receive higher MI scores based on the spoken corpus. That is, bigrams consisting of low-frequency words tend to receive higher MI scores regardless of the corpus used to calculate MI scores.

On the other hand, when bigram association strength was calculated based on t-scores that tend to inflate with high-frequency word combinations, different patterns were observed depending on the chosen corpus. L2 written responses as compared to L2 spoken responses were characterized by higher t-scores calculated using the academic corpus. In contrast, L2 spoken responses as compared to L2 written responses were characterized by higher t-scores calculated using the spoken corpus. A possible explanation for these different outcomes is that bigrams t-scores differed depending on the corpus chosen to calculate the scores. For example, bigrams that received higher t-scores calculated using the academic corpus include *such as, for example*, and *number of*, but their corresponding t-scores calculated using the spoken corpus were lower. In contrast, bigrams that received higher t-scores calculated using the spoken corpus include *this is, going to*, and *I mean*, but their corresponding t-scores calculated using the academic corpus were lower. Taken together, these results indicate that bigram t-scores may be helpful to distinguish L2 written and spoken output, but may not be reliable as measures of phraseological complexity given that t-score-based measures were found to be modality-sensitive.

The finding that L2 written output elicited greater lexical complexity than L2 spoken output corroborates previous research (e.g., Ellis and Yuan, 2005; Granfeldt, 2008; Kormos, 2014; Vasylets et al., 2017, 2019; Zalbidea, 2017). Going beyond previous studies, this study provides a newly found consistent pattern in terms of phraseological complexity, such that L2 written task performance tended to elicit more strongly associated bigrams as attested by higher bigram MI scores than L2 spoken task performance regardless of corpora (written and spoken) used to measure MI scores. Thus, based on previous studies that have found that MI-based measures can predict L2 writing and speaking performance (e.g., Bestgen and Granger, 2018; Kim et al., 2018; Paquot, 2019) and our findings that MI-based measures showed higher scores in L2 written output than L2 spoken output in a consistent, modality-insensitive manner, we advocate the notion that MI-based measures can be a proxy measure of phraseological complexity (Paquot, 2019).

Our findings that L2 written output has been found to be lexically and phraseologically more complex than L2 spoken output may be due to the nature of writing processes, where there is less time pressure and the written output is visible (Kormos, 2006; Williams, 2012; Tavakoli, 2014). That is, L2 learners may be able to spend more time formulating their ideas, accessing their L2 knowledge, and revising written output due to less time pressure and the visibility of the written output when writing (as compared when speaking). These findings accord with the notion that “writing constitutes a more favorable environment for the production of linguistically and propositionally complex discourse” (Vasylets et al., 2020, p. 185).

Finally, the results of the GLMM indicated the successful classification of L2 written and spoken responses with a high-level of accuracy (> 90%) using lexical and phraseological features. These results provide important and systematic difference between the written and the spoken modality in terms of lexical and phraseological features.

### 5.2. Research question 2: Characteristics of misclassified L2 written and spoken opinion responses

Research Question 2 investigated the characteristics of misclassified L2 written and spoken opinion responses in terms of lexical and phraseological features, L2 learners’ vocabulary knowledge, and raters’ judgments of L2 use.

First, the characteristics of L2 written opinion responses classified as spoken were examined. L2 written responses classified as spoken showed less lexical complexity (lower levels in lexical diversity, sophistication, and density) and less phraseological complexity (lower MI scores), which points to the characteristics of L2 spoken responses. To illustrate, an example of a written response classified as spoken (Example 1) and an example of a written response as written (Example 2) are presented in Table 8. These examples were written in response to the same prompt related to senior citizens. Example 1 (as compared to Example 2) has more characteristics of spoken responses with less lexical and phraseological complexity. The writer of example 1 also reported a lower L2 vocabulary scores and a lower L2 use score.

**Table 8:** Examples of L2 written opinion responses.

Example 1: A written response classified as spoken (218 words)	Example 2: A written response classified as written (219 words)
*First of all my position and my thought about this is simple. I think that this people need this. Why? Because it is a kind of practice to his minds.*	*I am writing you in concern about the article taken from the city times newspaper about the increasing desires of senior citizens for going back to school. I hope to say that I confidently agree with this new trend.*
*If we practice and do the mind work, we aren’t be more intelligents but we can prevent the Alzeimer for example. I mean if we have a lot of years old and we spend lots of time to rest, that will not be good for us.*	*First of all, going back to school will give them a new opportunity to create new relationships. Senior citizens may feel lonely. The majority of time since their families tend to get really into their jobs, their own children and problems, they rarely have time to visit their older family members.*
*So, now you know what I think, so if you ask me, what is the best way for senior citizens to spend their time? I’m going to answer you, that the best way is that this people must do activities because is healthy. Activities like memorized something, or do a light work, something that make they distract.*	*Moreover, they will feel more integrated to the world. The world has change a lot these last few years, and they being born in a different time difficulty their level of comprehension of the new technology being used nowadays. This new opportunity to learn will make them more independent.*
*On the other hand, the senior citizens whos worked all his life. I think that they should relax and have time to rest but at the same time do exercise for the mind, like play with maths or read a book.*	*On the other hand, the large generation gap with the younger student stay be a problem. Nowadays people want to do all really fast and having senior citizens with them may slow them down and make them angry since they do not have the rutine to deal with them.*
*In conclusion that senior citizens should be active doing little things because like this they can relax and have time to rest at the same time. This is going to be more healthy and it is going to help to his mind and memory.*	*In conclusion, senior citizens going back to school it is a great idea for their self-confidence but it will be better if they are supported of the young students.*

Example 1: HD-D = 0.785; Age of acquisition content words (no prompt words) = 5.113; Lexical density = 0.411; COCA spoken bigram MI = 1.446; L2 vocabulary score = 19; L2 use score = 2.5; Example 2: HD-D = 0.813; Age of acquisition content words (no prompt words) = 5.857; Lexical density = 0.488; COCA spoken bigram MI = 1.802; L2 vocabulary score = 26; L2 use score = 4; Due to confidentiality, the prompt is publicly unavailable.

We further examined one of the potential causes of producing these features, that is, L2 vocabulary knowledge. The results indicate that L2 vocabulary test scores tend to be lower for L2 learners whose written responses were classified as spoken than those whose written responses were correctly classified. This finding suggests that L2 learners’ lower levels in L2 vocabulary knowledge may have led to the production of lexically and phraseologically less complex written output in the L2, which in turn tended to have more characteristics of L2 spoken responses. In addition, given less time pressure during writing (Kormos, 2006; Williams, 2012; Tavakoli, 2014), it is possible that less complex lexical and phraseological features may have been partially due to L2 vocabulary knowledge itself, rather than the access to existing L2 vocabulary knowledge which was not much constrained during writing (as compared to speaking). Thus, our finding corroborates previous L2 writing research which has reported that lower L2 vocabulary knowledge is related to using less complex lexical features in L2 writing (e.g., Laufer and Nation, 1995; Henriksen and Danelund, 2015). The finding also supports the notion that lower levels of L2 lexical resources (as measured by an L2 vocabulary test) may hamper more complex lexical and phraseological encodings of L2 written message (Kormos, 2014).

In addition, we also examined the potential consequences of producing L2 written responses that had more characteristics of spoken responses. The *t*-test results indicate that L2 learners whose written responses were classified as spoken tended to receive lower scores in L2 use in their written responses than those whose written responses were correctly classified. These findings suggest that less complex lexical and phraseological features in L2 written output may have a negative influence on raters’ evaluation of L2 use. A possible explanation for this might be that the use of less complex lexical and phraseological features may be salient in the written output due to the visibility of writing, contributing to negative evaluation of L2 use. In addition, our finding gives support to previous studies which have reported that lower lexical and phraseological complexity (which are characteristics of spoken responses) are associated with poorer L2 writing performance (e.g., Kyle and Crossley, 2016; Bestgen and Granger, 2018; Kim et al., 2018; Paquot, 2019).

Next, the characteristics of L2 spoken responses classified as written were examined. L2 spoken responses classified as written showed greater lexical diversity, sophistication, and density, and less use of high-frequency word pairs based on a spoken corpus (as attested by lower t-scores based on the spoken corpus), which point to the characteristics of L2 written responses. An example of a spoken response misclassified as written (Example 3) and an example of a spoken response as spoken (Example 4) are presented in Table 9. These examples were responses to the same prompt related to achievement. Example 3 (as compared to Example 4) has more characteristics of written responses with greater lexical complexity and a lower bigram t-score based on the spoken corpus. Also note that the L2 vocabulary score and the L2 use score associated with Example 3 were comparable with Example 4’s scores.

**Table 9:** Examples of L2 spoken opinion responses.

Example 3: A spoken response classified as written (186 words)	Example 4: A spoken response classified as spoken (179 words)
*Not exactly failures, but to themselves, they might feel like their disappointment because they have not been able to achieve their greatest desires. So I do not consider them failures because they are rejected. They might have found something else or helped a lot of people, but I call them under-achievements, not really failures.*	*I do not think that they are failures because they all want to do and they are doing that.*
	*So I think that people are almost doing what they want and what they have to do.*
*Like success should be defined by how much their achievements are able to help people because theres almost about how many people a doctor can save or at least to a better health state or how many cures or at least things that can help ease the pain of people, a researcher can find, okay.*	*So if there are people that are doing that because they want to do it.*
	*I think that they are not a failure because you almost already getting one achievement about your life and getting some goal.*
*I found that being an engineer really lets people use their creativity and more of their imagination. It lets people think more freely, even when they are bound by some rules or there. The teacher is good because they help pave the way for future generations knowledge. It’s a good way to ensure the future.*	*And if they have to do what they need to do, I think that maybe they can failure in their life because they are not good with themself.*
	*A successful doctor.*
*If you work hard enough, a lot of times, things will work out eventually.*	*I think that not spending or not getting involved. Sort of so quickly with the things or maybe going.*
	*I think that maybe the doctors can help the people.*
*Society, at least here in Brazil, and some other parts of America, really do not recognize teachers.*	*And also can help themself not being involved too many times in works and I think that for me, that is a successful doctor because I want to do it that.*
	*I think that and sometimes, I would have a family and being a doctor, it cannot take me that time to pass with my family.*

Example 3: HD-D = 0.838; COCA spoken bigram T = 40.612: Age of acquisition content words (no prompt words) = 5.809; Lexical density = 0.443; L2 vocabulary score = 25; L2 use score = 5; Example 4: HD-D = 0.663; COCA spoken bigram T = 95.045: Age of acquisition content words (no prompt words) = 4.695; Lexical density = 0.323; L2 vocabulary score = 23; L2 use score = 4. Due to confidentiality, the prompt is publicly unavailable.

Furthermore, our results show that average L2 vocabulary test scores did not differ between L2 learners whose spoken opinion responses were misclassified as written and those whose spoken responses were correctly classified. These findings indicate that L2 learners’ vocabulary knowledge is less likely to relate to using lexical and phraseological features which is more in line with characteristics of written responses in spoken opinion responses. Instead, it might be the case that L2 learners whose spoken opinion responses were misclassified as written may have intentionally used more complex lexical complexity features and fewer bigrams consisting of high-frequency words in a spoken corpus in response to a spoken opinion task in order to better express their own thoughts and opinions or as a result of taking a high-stakes test that may activate greater genre awareness. More research, however, would be needed to explore potential causes of misclassified spoken responses.

In addition, average L2 use scores were not different between L2 learners whose spoken opinion responses were misclassified as written and those whose spoken responses were correctly classified. This finding suggests that the use of L2 lexical and phraseological features that were more in line with L2 written opinion responses in L2 spoken did not have a negative influence on raters’ evaluation of L2 use. A possible explanation for this finding is related to the genre-specific nature of tasks, such that both L2 written and spoken performances were opinion-giving tasks which required L2 learners to express their opinion and supporting details to a given topic by using language preferred in academic settings (Snow and Uccelli, 2009). That is, the use of lexical and phraseological features that were associated with L2 written opinion responses, which tend to show greater lexical and phraseological complexity and be preferred in academic settings, in L2 spoken opinion responses may have not negatively impacted raters’ evaluation of L2 use. In addition, this finding broadly supports the work of Biber et al. (2016), which found that L2 speaking opinion responses that showed more written lexical and grammatical characteristics tended to be higher rated. Similarly, L2 spoken opinion responses in our study that showed more characteristics of L2 written responses in terms of lexical and phraseological features were not negatively perceived by raters when evaluating L2 use.

## 6. Conclusion

The main goal of the current study was to examine differences in lexical and phraseological features between L2 written and spoken opinion performances. A secondary goal of this study was to investigate the characteristics of L2 written and spoken responses that were misclassified in terms of lexical and phraseological features, learners’ L2 vocabulary knowledge, and raters’ evaluation of L2 use. This study reports three main findings. First, L2 written and spoken responses were successfully classified using lexical and phraseological features. While much previous research has reported lexical differences between L2 written and spoken output (Ellis and Yuan, 2005; Granfeldt, 2008; Kormos, 2014; Biber et al., 2016; Vasylets et al., 2017, 2019; Zalbidea, 2017), to our knowledge, this study is the first to classify of L2 written and spoken responses based on lexical and phraseological features. Second, to our knowledge, this study is the first to explore the characteristics of incorrectly classified L2 written and spoken opinion responses. The findings indicate that the use of lexical and phraseological features of L2 spoken responses (i.e., less lexical and phraseological complexity) in L2 written responses may be partly due to L2 learners’ lower levels of L2 vocabulary knowledge, which also tend to penalize these learners in terms of raters’ judgments of L2 use. On the other hand, the use of lexical and phraseological features of L2 written responses (i.e., greater lexical and phraseological complexity) in L2 spoken responses does not tend to be related to L2 learners’ vocabulary knowledge or lead to lower scores by raters for L2 use. Thus, it seems reasonable to conclude that the use of greater lexical and phraseological complexity tends to lead to successful L2 written and spoken opinion responses, whereas the use of less lexical and phraseological complexity in L2 written opinion responses does not.

This study provides confirmatory evidence for distinguishing L2 written and spoken output. In line with the task-based language teaching literature, our findings indicate that L2 written and spoken tasks tend to elicit different levels of lexical and phraseological complexity (e.g., Vasylets et al., 2017, 2019; Zalbidea, 2017), supporting the notion that modality can be “perceived as an element of task complexity” (Kormos, 2014, p. 197). In addition, these findings corroborate research that supports the notion that writing and speaking skills are divisible (e.g., Bachman and Palmer, 1982; Sawaki et al., 2009; Kim and Crossley, 2020). This study also supports the hypothesis that writing has a greater potential for encouraging L2 learners to produce more linguistically complex language than speaking (Williams, 2012; Kormos, 2014; Vasylets et al., 2017, 2019). Given that the use of linguistically more complex language is indicative of L2 development (e.g., Housen et al., 2012; Ortega, 2012), pushing L2 learners to produce lexically and phraseologically more complex language in written output may foster deeper processing of the L2 system and develop learners’ interlanguage system (e.g., Skehan, 1996; Skehan and Foster, 1999).

Additionally, when considering differences between L2 written and spoken output, an important factor that needs to be considered is genre. While different genres may elicit different levels of L2 use (Berman and Ravid, 2009), our findings indicate consistent patterns across modalities in the opinion-giving genre, such that greater lexical and phraseological complexity tend to be preferred (or, at least, not be penalized) for both writing and speaking. This finding contributes to expanding our current understanding of the relationships among genres, modalities, and lexical and phraseological complexity, such that greater lexical and phraseological complexity tends to lead to successful language use in a modality-insensitive manner in the academic genre of giving one’s opinion.

The findings of this study also suggest two main pedagogical implications. First, if the focus of instruction is to elicit and consolidate more lexically and phraseologically complex L2 use in an opinion-giving task across modalities, choosing a written task is likely more effective than choosing a spoken one. Second, if L2 learners produce L2 written opinion output that shows more characteristics of spoken language use (i.e., less lexical and phraseological complexity), they can be instructed to use produce more complex lexical and phraseological features and improve their L2 vocabulary knowledge. In contrast, if L2 learners produce L2 spoken opinion output that shows more characteristics of written language use (i.e., greater lexical and phraseological complexity), interventions may not be needed because such characteristics do not tend to lead to lower levels of L2 use scores.

There are limitations to this study that can be addressed in future research. First, this study focused on lexical complexity only as part of the framework of complexity, accuracy, and fluency (CAF) that characterize L2 performance. Future studies could examine various CAF measures when comparing L2 written and spoken output (*cf.* Vasylets et al., 2020). Second, we used one type of task (i.e., opinion-giving). Future studies could examine and compare other types of tasks across modalities. Third, we used a relatively small corpus. Partly due to this, we had a small number of misclassified samples for comparisons. Future studies could use a larger dataset to better generalize the findings of this study. Fourth, we did not control for individual difference variables, such as L1 backgrounds and exposure to oral and written L2s, and L2 learning experiences. Last, we examined L2 written and spoken output, but not online processes of how modality impacts L2 learners’ production of L2 output. Future studies could examine how modality might affect L2 learning processes, such as noticing and restructuring (i.e., development of the L2 system).

## Data availability statement

The original contributions presented in the study are included in the article/supplementary materials, further inquiries can be directed to the corresponding author.

## Author contributions

MK and SC: conceptualization, data curation, analysis, and methodology. MK: writing–original draft. SC: writing–review and editing. All authors contributed to the article and approved the submitted version.

## Funding

For open access publication fees, this work was supported by the Japan Society for the Promotion of Science (JSPS) KAKENHI Grant Number 20 K13119. This work reports on research funded through Michigan Language Assessment’s Spaan Research Grant Program, 2017.

## Conflict of interest

The authors declare that the research was conducted in the absence of any commercial or financial relationships that could be construed as a potential conflict of interest.

## Publisher’s note

All claims expressed in this article are solely those of the authors and do not necessarily represent those of their affiliated organizations, or those of the publisher, the editors and the reviewers. Any product that may be evaluated in this article, or claim that may be made by its manufacturer, is not guaranteed or endorsed by the publisher.

## Footnotes

^1^In this study, we used the term task to refer to a communicative task in classroom or assessment contexts.

^2^Due to confidentiality, the prompts for writing and speaking tasks used in this study are not publicly available.
